# Should we ignore SARS-CoV-2 disease?

**DOI:** 10.1017/S0950268824000487

**Published:** 2024-03-20

**Authors:** Igor Nesteruk

**Affiliations:** Institute of Hydromechanics, National Academy of Sciences of Ukraine, Kyiv, Ukraine

**Keywords:** Brazil, China, COVID-19 epidemic dynamics in USA, COVID-19 mortality, COVID-19 pandemic, endemic characteristics of SARS-CoV-2, France, Germany, India, Italy, Japan, mathematical modelling of infectious diseases, South Korea, statistical methods, UK

## Abstract

Current World Health Organization (WHO) reports claim a decline in COVID-19 testing and reporting of new infections. To discuss the consequences of ignoring severe acute respiratory syndrome coronavirus-2 (SARS-CoV-2) infection, the endemic characteristics of the disease in 2023 with the ones estimated before using 2022 data sets are compared. The accumulated numbers of cases and deaths reported to the WHO by the 10 most infected countries and global figures were used to calculate the average daily numbers of cases *DCC* and deaths *DDC* per capita and case fatality rates (*CFRs = DDC/DCC*) for two periods in 2023. In some countries, the *DDC* values can be higher than the upper 2022 limit and exceed the seasonal influenza mortality. The increase in *CFR* in 2023 shows that SARS-CoV-2 infection is still dangerous. The numbers of COVID-19 cases and deaths per capita in 2022 and 2023 do not demonstrate downward trends with the increase in the percentages of fully vaccinated people and boosters. The reasons may be both rapid mutations of the coronavirus, which reduced the effectiveness of vaccines and led to a large number of re-infections, and inappropriate management.

## Introduction

The high numbers of circulating severe acute respiratory syndrome coronavirus-2 (SARS-CoV-2) variants [1–3] and re-infected people [4–6], the lack of decreasing trends in the global numbers of deaths [7], and the expected very long duration of the Omicron wave [8] caused an assumption that the pathogen will circulate forever, which was supported by a mathematical model [9]. Some endemic characteristics of the SARS-CoV-2 disease were estimated in [9] with the use of global accumulated numbers of COVID-19 cases *V* and deaths *D*, registered in [10] from 1 January 2022 to 6 December 2022.

In this study, we will try to check the estimations presented in [9] with the use of data reported by the World Health Organization (WHO) in 2023 [7]. Unfortunately, currently ‘reported cases do not accurately represent infection rates due to the reduction in testing and reporting globally’ [11]. In particular, in the period from 31 July to 27 August 2023, only 39% of countries reported at least one case [11]. Of the 10 countries with the highest figures of accumulated COVID-19 cases, only four (Italy, the UK, India, and China) have reported new cases during the last 28 days of 2023 [7]. Nevertheless, we will try to analyse recent trends for average daily numbers of cases *DCC* and deaths *DDC* per million and the case fatality risk (CFR), and the influence of vaccination levels (the percentages of fully vaccinated people *VC* and boosters *BC* listed in [10]).

## Materials and methods

We will use the accumulated numbers of laboratory-confirmed COVID-19 cases *V_i_* and deaths *D_i_* for 10 countries with the highest figures and the world (*i* = 1, 2, …, 11) listed by the WHO [7] (version of file updated on 14 September 2023). Chinese figures include Mainland China, Taiwan, Hong Kong, and Macao. 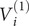 , 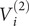 , and 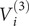 correspond to the numbers of cases accumulated on 31 December 2022, 15 May 2023, and 10 September 2023, respectively. 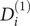 , 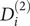 , and 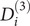 correspond to the accumulated numbers of deaths (see Table 1). The populations *N_i_* of regions (as of 14 September 2023 [12]) are also listed in Table 1.

**Table 1.** Accumulated numbers of COVID-19 cases and deaths and population volumes in 2023

No., I	Country or region	Population in millions, as of 14 September 2023 [12], *N_i_*	Accumulated numbers of confirmed COVID-19 cases [7]			Accumulated numbers of COVID-19-related deaths [7]		
			31 December 2022, 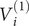	15 May 2023, 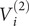	10 September 2023, 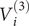	31 December 2022, 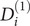	15 May 2023, 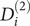	10 September 2023, 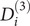
1	USA	340.4	99,411,696	103,436,829	103,436,829	1,082,456	1,127,152	1,127,152
2	China	1,425.6	84,925,042	99,238,850	99,238,850	52,544	120,896	120,896
3	India	1,431.3	44,678,384	44,981,475	44,997,710	530,702	531,778	532,027
4	France	67.8	37,989,547	38,898,196	38,997,490	161,667	167,357	167,985
5	Germany	83.3	37,241,936	38,419,325	38,437,756	165,738	174,682	174,979
6	Brazil	216.7	36,302,415	37,511,921	37,717,062	693,734	702,116	704,659
7	South Korea	51.8	29,059,273	31,415,280	34,571,873	32,272	34,623	35,934
8	Japan	123.2	29,105,070	33,803,572	33,803,572	57,513	74,694	74,694
9	Italy	58.8	25,168,438	25,833,895	25,977,012	184,792	190,138	191,370
10	UK	67.8	24,194,538	24,608,306	24,704,113	216,112	227,329	229,089
11	The world	8,060.5	729,473,239	765,989,469	770,563,467	6,719,111	6,937,164	6,957,216

To calculate the average daily characteristics of *DCC* and *DDC,* we will use two periods, 1 January–10 September 2023 (*T_1_* = 253 days) and 16 May–10 September 2023 (*T_2_* = 119 days), and simple formulas:

_(1)_

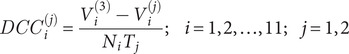




_(2)_

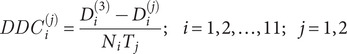

The CFR values corresponding to the same periods can be calculated as follows:

_(3)_

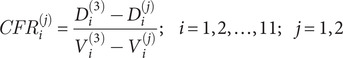



## Results and discussion

**Figure 1.**
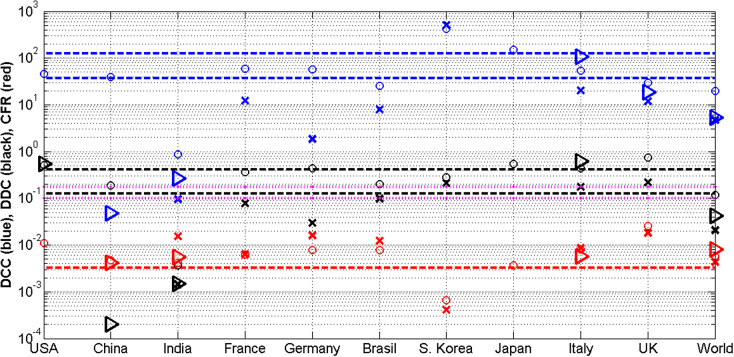
Results of calculations (markers) of the average daily numbers of cases *DCC* (blue) and deaths *DDC* (black) per million and case fatality risks (*CFRs*) (red) for three different periods in 2023 and comparison with the values estimated in [9] (dashed lines) and flu mortality (dotted magenta lines). The characteristics, corresponding to the long period (1 January–10 September 2023; *T_1_* = 253 days), are shown by ‘circles’; to the short period (16 May–10 September 2023; *T_2_* = 119 days), by ‘crosses’; and to the last 28 days of 2023, by ‘triangles’.

The results of calculations are presented in Table 2 and Figure 1. Blue, black, and red markers correspond to the values of *DCC, DDC,* and *CFR*, respectively. The characteristics, corresponding to the long period (1 January–10 September 2023; *T_1_* = 253 days), are shown by ‘circles’; to the short period (16 May–10 September 2023; *T_2_* = 119 days), by ‘crosses’; and to the last 28 days of 2023, by ‘triangles’. Zero values and cases when CFR cannot be calculated are not shown in Figure 1.

According to the endemic characteristics of the SARS-CoV-2 disease estimated in [9] with the use of *V* and *D* values, registered in [10] from 1 January 2022 to 6 December 2022, the global numbers of new daily cases will range between 300 thousand and one million and daily deaths between one and 3.3 thousand. Dividing these values by the world population of 8,060.5 million [12], we can calculate lower and upper limits per capita: for the global average number of daily cases per million, DCC* = 37.2 and DCC^**^ = 124.1, and for the global average number of COVID-19-related deaths per million, DDC* = 0.124 and DDC^**^ = 0.409. The global value of case fatality risk CFR* = 0.0033 for the period from 1 January 2022 to 6 December 2022 [9]. These values are represented by dashed lines: blue for DCC* and DCC^**^, black for DDC* and DDC^**^, and red for CFR*. For comparison, the dotted magenta lines represent the limits DDC*_(flu)_ = 0.1 and DDC^**^_(flu)_ = 0.18 for the global average daily number of deaths per million related to the seasonal flu calculated in [13] using the figures registered in 2002–2011 [14].

It can be seen that the average values of new daily cases per capita corresponding to the long period ( 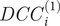 , blue ‘circles’) are mostly close to the estimation DCC* = 37.2 (the lower blue line). The value 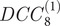 corresponding to Japan is slightly higher than DCC^**^ = 124.1. In 2023, South Korea registered much more cases than the global average value in 2022 (compare the corresponding blue ‘circle’ and ‘cross’ with the upper blue line). This country applied a zero-COVID strategy that ‘involves using public health measures such as contact tracing, mass testing, border quarantine, lockdowns, and mitigation software in order to stop community transmission of COVID-19 as soon as it is detected’ [15]. Due to this, the zero-COVID countries have registered much more cases per capita *CC* and achieved much lower numbers of deaths per capita *DC* and *CFR* values in comparison with other countries and regions [16].

The average daily numbers of cases per capita corresponding to the short period are zero for the USA, China, and Japan (not shown in Figure 1) or much lower than the estimation DCC* = 37.2 (compare blue ‘crosses’ and the lower blue line). There are two possible reasons for this result. As mentioned below, more and more countries have reduced testing and stopped updating the COVID-19 statistics [10, 11]. On the other hand, the short period corresponds to the summer time in the most infected countries. During the winter period (the last 28 days of 2023), only Italy, the UK, India, and China have reported new cases (175.9 K, 35.8 K, 11 K, and 1.9 K, respectively, with the global figure 1,189,927 [7]). Dividing these values by 28 and the volumes of populations listed in Table 1, we can obtain the corresponding average daily numbers of new cases per million *DCC* = 106.8, 18.9, 0.27, and 0.048, respectively, with the global figure 5.3 (see blue ‘triangles’). The Italian figure 106.8 approaches the upper limit DCC^**^ = 124.1 and shows that new severe COVID-19 pandemic waves are still possible.

Very low numbers of cases and deaths per capita registered in India look like exclusion (compare the corresponding blue and black ‘circles’, ‘crosses’, and ‘triangles’ with the blue and black lines) connected with the low testing level typical for low-income countries (see [17]). The ratio of the number of tests to the number of cases *DTS* will allow us to understand the situation. High *DTS* values mean that many people surrounding the detected infectious patient (e.g. family members, colleagues, neighbours) were tested and isolated (this causes a decrease in the number of new infections, i.e. *DCC*). For example, very high tests per case ratios (*DTS* > 100) in Hong Kong in 2020 and 2021 allowed controlling the COVID-19 epidemic completely [18] (the smoothed daily numbers of new cases per million were less than 20 [10]). The average daily test per case ratio increases for countries with low *DCC* figures [13]. For example, *DTS* values in 2022 were equal to 26.9 (the UK), 39.4 (India), 4.3 (South Korea), and 1.95 (Japan) and demonstrate that the probability of missing an infectious person due to the lack of tests is much higher in Japan or South Korea than in India [13]. During the severe pandemic wave in Japan in the summer of 2022, the testing levels probably were not enough to confirm COVID-19 in all patients with symptoms [19].

Very low *DCC* and *DDC* values registered in India could be the result of large numbers of asymptomatic undetected cases. The ratio of real and registered cases (visibility coefficient 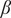 ) could be rather high due to large numbers of asymptomatic COVID-19 patients [20] and avoiding testing by infectious people with moderate symptoms. An experimental estimation of the visibility coefficient 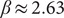 was obtained in [21] from the results of total testing in Slovakia on 31 October–7 November 2020 [22]. The generalized susceptible–infected–removed (SIR) model and algorithms of their parameter identification [19, 21] yielded theoretical 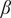 values from 3.7 to 20.4 for Ukraine [21, 23] and 5.4 for Qatar [24] in different periods of the pandemic. The rapid decrease in *DCC* values in 2023 (see Table 2 or compare blue ‘circles’ and ‘crosses’) illustrates the increase in visibility coefficients due to the sharp reduction in the public interest in SARS-CoV-2 infection and testing levels. In particular, the huge growth of 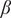 values makes control of endemic characteristics of SARS-CoV-2 infection [9] impossible. The lack of appropriate testing did not allow proper detection of the first cases [25, 26], which probably appeared in August 2019 [25].

**Table 2.** Results of calculations of the average daily numbers of cases and deaths and case fatality rates for two different periods in 2023

No., *I*	Country or region	Average daily numbers of new COVID-19 cases per million, *DCC_i_* for different periods in 2023		Average daily numbers of COVID-19-related deaths per million, *DDC_i_* for different periods in 2023		Case fatality rates, *CFR_i_* for different periods in 2023	
		1 January−10 September, 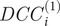	15 May–10 September, 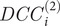	1 January−10 September, 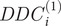	15 May–10 September, 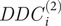	1 January−10 September, 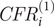	15 May–10 September, 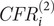
1	USA	46.7	0	0.519	0	0.0111	–
2	China	39.7	0	0.190	0	0.00477	–
3	India	0.88	0.095	0.00366	0.00146	0.00415	0.0153
4	France	58.8	12.3	0.368	0.0778	0.00627	0.00632
5	Germany	56.7	1.86	0.438	0.03	0.00773	0.0161
6	Brazil	25.8	7.95	0.199	0.0986	0.00772	0.0124
7	South Korea	420.6	512.1	0.279	0.213	0.000664	0.000415
8	Japan	150.7	0	0.551	0	0.00366	–
9	Italy	54.4	20.5	0.442	0.176	0.00814	0.00861
10	UK	29.7	11.9	0.757	0.218	0.0255	0.0184
11	The world	20.1	4.77	0.117	0.0209	0.00579	0.00438

Assuming that the visibility coefficients are proportional to the fraction of asymptomatic patients, which is much higher in children [20], and that younger populations have fewer clinical cases per capita [27], the population age was taken into account in the statistical studies [13, 16]. Rather strong correlations between the median age *A* and *DCC* and *DDC* values registered in 2022 and 2023 in 31 countries and regions were revealed in [13] (a similar strong trend for *CC* and *DC* values vs. *A* was demonstrated in [16]). One-year increment in the median age yields the average increase in *DCC* values by 39.8 in 2022 and by 5.8 in 2023 (for *DDC* values by 0.0799 in 2022 and by 0.0263 in 2023) [13]. For example, taking into account the median age difference (*A* = 28.7 in India and 30.5 for the world [28, 29]), we can calculate the differences in *DCC* (10.4) and *DDC* (0.0473) expected in 2023. These values are located between corresponding differences for the short and long periods of 2023 (see Table 2). Thus, much lower numbers of COVID-19 cases and deaths per capita registered in India or Africa [7, 10, 13, 16, 17, 30] are a result of the huge amount of asymptomatic (and unrevealed) infections due to the younger populations.

It can be seen that average values of daily deaths per capita corresponding to the long period ( 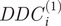 , black ‘circles’) are mostly located between the limits DDC* and DCC^**^ (black lines). It means that these estimations (obtained in [9] with the use of 2022 data sets) still correspond to the real number of deaths. Zero or much lower numbers of deaths during the short period ( 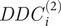 , black ‘crosses’) probably reflect the reduction in testing and a seasonal decrease in mortality. During the winter period (the last 28 days of 2023), only the USA, Italy, India, and China have reported the new COVID-19-related deaths (5.2 K, 1 K, 61, and 8, respectively, with the global figure 9,575 [7]). Then, *DDC* values were equal to 0.55, 0.61, 0.0015, and 0.0002, respectively, with the global figure 0.042 (see black ‘triangles’). The US and Italian figures significantly exceed the upper limits of DDC^**^ = 0.409 for COVID-19 in 2022 and DDC^**^_(flu)_ = 0.18 for the global average flu mortality. The mortality related to the flu and pneumonia is much lower in these countries (age-standardized average daily numbers of deaths per million ( *ASDDC* ) can be estimated as 0.24 and 0.17, respectively [31]). *ASDDC* values for China, Japan, the UK, and South Korea vary from 0.31 to 0.69 [31] and are lower than *DDC^(1)^* figures only in Korea and China (see Table 2). The *ASDDC* value for India (0.97) is much higher than COVID-19-related mortality, probably due to the difference in age distributions (neglected in *DDC* calculations).

Since the USA has reported only the number of deaths during the last 28 days of 2023, we have calculated the corresponding *CFR* values only for Italy (0.0057), India (0.0055), China (0.0042), and the global figure (0.0080) (see red ‘triangles’). Much higher CFRs in 2023 (compare red markers with the red line corresponding to 2022; South Korea is only one exception) show that SARS-CoV-2 infection is still dangerous despite increasing vaccination level (more than 13.5 billion doses have been administered as of 31 August 2023 [11]). In different periods of the pandemic, *CFR* demonstrated decreasing trends with the increase in *VC* and *BC* [13, 17, 32, 33].

To remove the age structure influence, current age-standardized *CFR* (*ASCFR*) values for the COVID-19 pandemic can be calculated and compared with the ones corresponding to 2022. For the period before 6 April 2021, *ASCFR* values in the UK, South Korea, Japan, the USA, Italy, and Germany were 2–3 times higher than non-standardized *CFR* values [34]. In 2022 and 2023, *CFR* unexpectedly demonstrated decreasing trends with the increase in the median age of population [16]. Since *CFR* is the probability to die for a person tested positive, it depends not only on the individual state of health and immunity (related to age and vaccinations) but also to a large extent on the speed and quality of medical care, which are better in countries with higher incomes (and older populations). As of 1 August 2022, the *DC* and *CFR* values decreased for richer European countries [17]. Probably, the better medical care and higher vaccination level in high-income countries help reduce *CFR* despite older populations.

In 2023, the higher *CFR* values can also be a result of reduction in testing levels. Non-linear correlation demonstrated that the CFR drastically increased at low numbers of tests per capita even in 2022 [13]. In 2023, when people paid attention to severe cases only and make tests correspondingly, *CFR* values can increase (reducing the denominator in formula (3) can be much larger than reducing the nominator). Therefore, one should probably not be afraid of a significant increase in *CFR* in 2023.

Of much greater concern is the fact that COVID-19 mortality is still high and calls into question of the effectiveness of vaccinations. Probably, the rapid mutations of coronavirus [1–3] reduce the effectiveness of vaccines and cause high numbers of re-infections [4–6]. The trends for *DCC* and *DDC* values and/or corresponding accumulated numbers of cases *CC* and deaths *DC* per capita versus percentages of vaccinations *VC* and boosters *BC* were investigated for different periods of the COVID-19 pandemic (see examples in [13, 17–19, 23, 32, 33, 35] and Table 3). In 2021 and 2022, severe pandemic waves (with very high *DCC* and *DDC* numbers) occurred in Israel, Japan, Hong Kong, and other countries with high vaccination levels (see lines 1, 2, 5, and 10 in Table 3 and [18, 19, 23, 33, 35]). With the use of non-linear correlation, very strong growing trends with the increase in *VC* were revealed for *CC* and *DC* values accumulated in all European and African countries from the pandemic outbreak until 1 August 2022 [17]. The same trends demonstrate *DCC* and *DDC* calculated in [13] for 2022 and the period from 1 January 2023 to 10 September 2023 using Johns Hopkins University (JHU) data sets [10] for 34 countries and regions. At the confidence level of 0.01, one-per cent increment of *VC* causes the average of 13.3 increase in *DCC* in 2022 and 2.7 in 2023; the increases in *DDC* are 0.026 and 0.011, respectively; and one-per cent increment of *BC* causes the average of 14.7 increase in *DCC* in 2022 and 1.7 in 2023; the increases in *DDC* are 0.025 and 0.0068, respectively [13]. Since percentages of vaccinations increase with the growth of the population age, the corrected variations of *VC* and *BC* were considered [13]. Nevertheless, no decreasing trends for *DCC* and *DDC* values versus corrected variations were revealed. However, only further statistical and comparative studies can definitively put an end to the very important question of vaccination effectiveness during the COVID-19 pandemic.

**Table 3.** Trends for accumulated cases (*CC)* and deaths (*DC)* per capita *and* average daily new cases (*DCC)* and deaths (*DDC)* per capita vs. percentages of fully vaccinated people (*VC)* and boosters (*BC)* in different periods of the COVID-19 pandemic

No.	Characteristics, source of data, method of 7-day smoothing	Country/region, period	Trend with the increase in *VC*, confidence levels (*p*-values)	Trend with the increase in *BC*, confidence levels (*p*-values)	Reference
1	*DCC*, [10], [25]	Qatar, March and April 2021	Increasing at *VC* by around 50%	–	[24]
2	*DCC* and *DDC*, [10], [25]	Israel, June–August 2021	Increasing at *VC* by around 60%	–	[23]
3	*DCC*, [10]	Europe+10 more countries and regions, 1 September 2021	No correlation, *p* = 0.1	–	[32]
4	*DDC*, [10]	Europe+10 more countries and regions, 1 September 2021	Decreasing, *p* = 0.1	–	[32]
5	*DCC* and *DDC*, [10], [25]	Argentina, Brazil, the USA, the UK, EU, and Australia, November 2021–January 2022	Increasing at *VC* higher than 50%	Increasing at *BC* 1%–54%	[35]
6	*DCC*, [10]	Europe+15 more countries and regions, 1 February 2022	Increasing, *p* = 0.01	Increasing, *p* = 0.01	[33]
7	*DDC*, [10]	Europe+15 more countries and regions, 1 February 2022	Decreasing, *p* = 0.05	Decreasing for Europe, *p* = 0.001; no correlation for Europe+15, *p* = 0.1	[33]
8	*CC*	Europe + Africa, accumulated as of 1 August 2022	Increasing, *p* = 0.001	–	[17]
9	*DC*	Europe + Africa, accumulated as of 1 August 2022	Increasing, *p* = 0.001	–	[17]
10	*DCC* and *DDC*, [10], [25]	Japan, July–August 2022	Increasing at *VC* higher than 82%	Increasing at *BC* 68%–87%	[19]
11	*DCC* and *DDC*	34 countries and regions, 2022 and 1 January–10 September 2023	Increasing, *p* = 0.01	Increasing, *p* = 0.01	[13]

Improper organizational measures may also have contributed to the failure of the vaccination campaign. Many vaccinated people in countries with high *VC* and *BC* values were allowed to visit crowded places and travel despite they could spread the infection. Overcrowding in hospitals during vaccinations in many countries (Ukraine is an example) could contribute to the spread of the infection too. Here, the change in trends for *DCC* and *DDC* values in European countries calculated with 7-day smoothing [10] is indicative. As of 1 September 2021, no correlation versus *VC* was revealed for *DCC* values and DDC figures showed a decreasing trend [32], while as of 1 February 2022, *DCC* showed an increasing trend versus *VC* and *BC* (one-per cent increment in *VC* and *BC* caused, respectively, 40.9 and 38.7 increase in *DCC* values; *DDC* values demonstrated decreasing trends versus *VC* and *BC* [33]). *CC* and *DC* values accumulated in 15 European countries with the highest testing level from the outbreaks until 1 August 2022 demonstrated no decreasing trends versus *VC* at the confidence level of 0.05 [17].

## Conclusions

The endemic characteristics of SARS-CoV-2 infection in 2023 agree with the ones estimated in [9] with the use of 2022 data sets, but lower testing and reporting levels have to be taken into account. The average values of daily deaths per million still can be higher than the upper limit of 0.41 and exceed seasonal influenza mortality.

An increase in the *CFRs* in 2023 shows that SARS-CoV-2 infection is still dangerous. Since the numbers of COVID-19 cases and deaths per capita in 2022 and 2023 do not demonstrate downward trends with the increase in the percentages of fully vaccinated people and boosters, the vaccination campaign probably was not effective. The reasons may be both rapid mutations of the coronavirus, which reduced the effectiveness of vaccines and led to a large number of re-infections, and inappropriate management.

## Data Availability

All data generated or analysed during this study are included in this text.

## Clarification point

No humans or human data were used during this study.
